# Observing the Central Arctic Atmosphere and Surface with University of Colorado uncrewed aircraft systems

**DOI:** 10.1038/s41597-022-01526-9

**Published:** 2022-07-23

**Authors:** Gijs de Boer, Radiance Calmer, Gina Jozef, John J. Cassano, Jonathan Hamilton, Dale Lawrence, Steven Borenstein, Abhiram Doddi, Christopher Cox, Julia Schmale, Andreas Preußer, Brian Argrow

**Affiliations:** 1 Cooperative Institute for Research in Environmental Sciences, University of Colorado Boulder, Boulder, Colorado USA; 2 Physical Sciences Laboratory, National Oceanic and Atmospheric Administration, Boulder, Colorado USA; 3 Integrated Remote and In Situ Sensing, University of Colorado Boulder, Boulder, Colorado USA; 4 National Snow and Ice Data Center, University of Colorado Boulder, Boulder, Colorado USA; 5 Department of Atmospheric and Oceanic Sciences, University of Colorado Boulder, Boulder, Colorado USA; 6 Department of Aerospace Engineering Sciences, University of Colorado Boulder, Boulder, Colorado USA; 7 Extreme Environments Research Laboratory, École Polytechnique Fédérale de Lausanne, Sion, Switzerland; 8 Department of Environmental Meteorology, University of Trier, Trier, Germany; 9 Helmholtz Centre for Polar and Marine Research, Alfred-Wegener-Institute, Bremerhaven, Germany

**Keywords:** Atmospheric dynamics, Cryospheric science

## Abstract

Over a five-month time window between March and July 2020, scientists deployed two small uncrewed aircraft systems (sUAS) to the central Arctic Ocean as part of legs three and four of the MOSAiC expedition. These sUAS were flown to measure the thermodynamic and kinematic state of the lower atmosphere, including collecting information on temperature, pressure, humidity and winds between the surface and 1 km, as well as to document ice properties, including albedo, melt pond fraction, and open water amounts. The atmospheric state flights were primarily conducted by the DataHawk2 sUAS, which was operated primarily in a profiling manner, while the surface property flights were conducted using the HELiX sUAS, which flew grid patterns, profiles, and hover flights. In total, over 120 flights were conducted and over 48 flight hours of data were collected, sampling conditions that included temperatures as low as −35 °C and as warm as 15 °C, spanning the summer melt season.

## Abstract (summary)

**Table:** 

Measurement(s)	NetCDF files include full description of all variables
Technology Type(s)	Uncrewed Aircraft Systems

## Background & Summary

The last several decades have seen tremendous changes in the Arctic Environment^1,2^. Warming temperatures^3,4^, thinning and retreating sea ice^5,6^ and evolving ocean conditions^7,8^ all indicate a changing Arctic climate and transition to a new climatic state. Such changes are thought to have significant influence on global climate^9,10^, and at the same time result in opportunities for enhanced commercial activity in this region^11–13^. To observe and document the status of the “new Arctic”, the MOSAiC (Multidisciplinary drifting Observatory for the Study of Arctic Climate) expedition^14^ was conducted between September 2019 and October 2020. Under MOSAiC, the research icebreaker *R/V Polarstern*^15^ was frozen into the sea ice and allowed to drift across the central Arctic carrying an international team of scientists. Collectively, these participants observed many aspects of the Arctic system, including the ocean^16^, ice^17^, atmosphere^18^, ecosystems, and biogeochemistry.

**Fig. 1:**
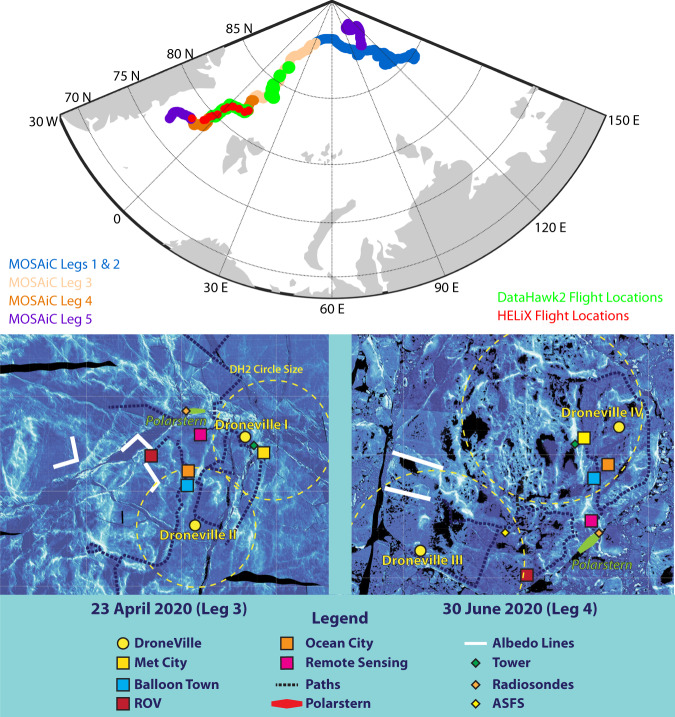
A map illustrating flight locations, along with floe maps illustrating the location of UAS operations relative to other MOSAiC assets during legs 3 and 4. The operating area map (top) shows the MOSAiC drift track, broken down by leg, along with the launch locations of the two platforms for all flights. The floe maps (bottom) are derived from helicopter-based airborne laser scanner data (L. von Albedyll and S. Hendricks, pers. comm.) of the sea ice collected on April 23, 2020 (left) and June 30, 2020 (right). The UAS sites are labeled as “Droneville”, and a circle illustrating the approximate size of a DataHawk2 orbit is included around each location. Also shown are the locations of key sensors for comparisons conducted in this manuscript, including the ASFS, radiosondes, and the met tower.

To support MOSAiC and provide unique observational perspectives on the Arctic atmosphere and surface to help address key questions on the changes highlighted above, a research team from the University of Colorado Boulder and NOAA Physical Sciences Laboratory deployed to operate a fleet of small uncrewed aircraft systems (sUAS). The deployment of sUAS to the central Arctic for a six-month window from late winter through summer represents a significant technological addition to MOSAiC, extending observational coverage beyond what was achieved during previous studies in this region (e.g., SHEBA^19^). These platforms were deployed to achieve various observational goals, including enhanced vertical profiling of the lower atmosphere around *Polarstern*, capturing spatiotemporal variability of the atmosphere and underlying surface around *Polarstern*, and documenting the influence of the surface on lower-atmospheric evolution. The primary operating areas for these sUAS are shown in Fig. 1. Specific observational goals included: high-resolution profiling of the thermodynamic and kinematic states of the lower atmosphere, providing detailed observations of turbulence between the surface and one kilometer altitude; high spatial-resolution observing of the variability of broadband surface reflectivity (albedo) and its evolution throughout the melt season; and mapping the make-up of the ice surface and how it evolves over time, including detailed information on spatial scales ranging between several centimeters and several hundred meters. The vertical profiling efforts were developed to understand the structure of the lower atmosphere, including the properties of the atmospheric boundary layer, lower atmosphere influence on cloud formation and lifecycle, the impact of warm air intrusions in the region, and the drivers of turbulence, and were meant to expand the insights gained from the multitude of surface-based remote and *in-situ* sensing systems deployed during MOSAiC. Additionally, targeted surveys to measure thermodynamic and kinematic state were designed to obtain information on the influence of leads in the sea ice surface on the overlying atmosphere. Similarly, the surveys of albedo and surface type complement efforts to document the evolution of these properties by other teams and sensors operating on the ice surface and by helicopter. These surveys were designed to support studies of surface evolution and its connection to atmospheric and oceanic events, bridging the scales between observations collected from the ice, by air, and from satellite platforms.

To achieve these objectives, three different sUAS platforms were deployed, including the DataHawk2, RAAVEN and HELiX. Flights conducted by these platforms were to take place during legs 3–5 of MOSAiC, spanning between late February and mid-August 2020. The original plan was to deploy only DataHawk2 during leg 3, when atmospheric conditions were expected to be the coldest and the surface was expected to be relatively homogeneous, and then additionally deploy RAAVEN and HELiX during legs 4 and 5. Due to the COVID-19 pandemic, MOSAiC logistics were modified, extending leg 3 and eliminating one of the remaining three re-supply actions. Additionally, *Polarstern* was removed from the ice during the transition between legs 3 and 4 (mid-May to mid-June). Together, these modifications significantly impacted sUAS sampling, reducing the time available for RAAVEN and HELiX operations, and introducing a data gap of approximately one month during the leg transition. Unfortunately, RAAVEN additionally suffered from a data-logging system malfunction that could not be remedied during the drift, resulting in the grounding of that aircraft without any science flights being conducted. Therefore, the DataHawk2 and HELiX carried the sampling load, completing a total of 121 flights for a combined 48.43 flight hours, with DataHawk2 flights taking place between 23 March and 26 July 2020, and HELiX flights taking place between 15 June and 6 August 2020. These two systems and the data they produced are the focus of the current manuscript.

## Methods

**Fig. 2:**
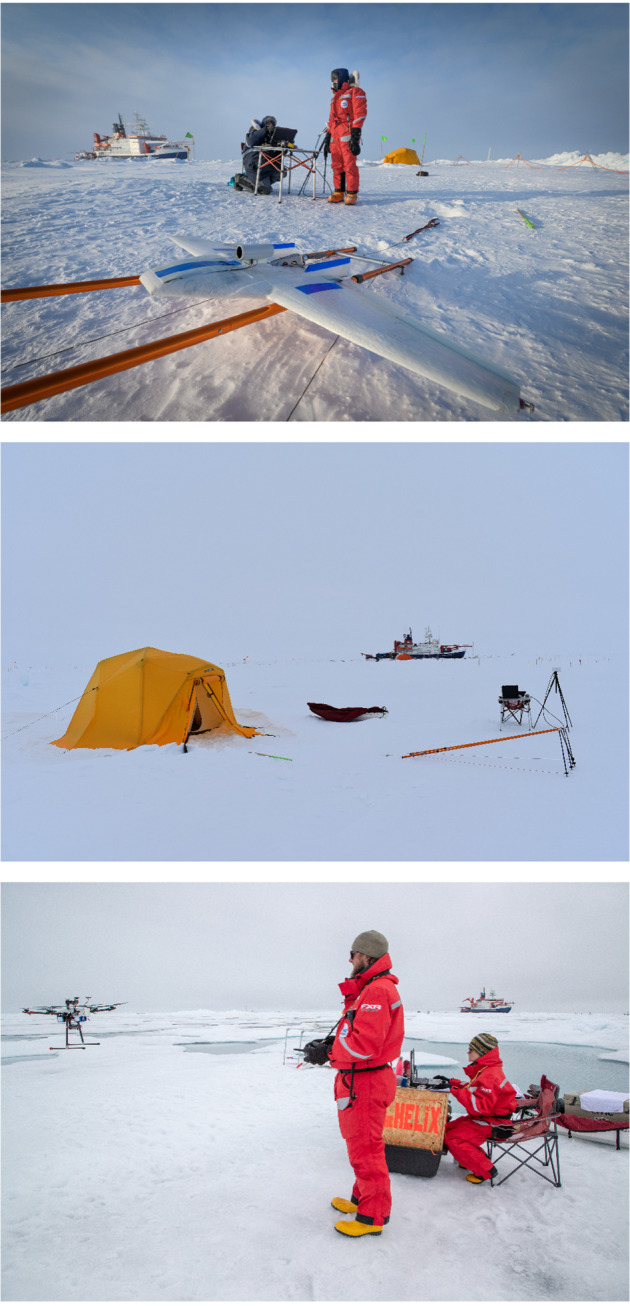
Photographs depicting the operating sites used during MOSAiC. From top to bottom, these include Droneville I (photo: Delphin Ruche) and Droneville II (photo: Gina Jozef), both depicted during leg 3 of MOSAiC, and Droneville III (photo: Lianna Nixon), as seen during leg 4 of the campaign. Note the *Polarstern* in each photograph, for perspective on the distance from the vessel. Informed consent to publish individual likeness was obtained.

The two sUAS systems used for the development of this dataset have different characteristics, and data collected by each are processed differently. Additionally, each aircraft type has a unique instrument payload and processing techniques. Both platforms were operated from the ice near Polarstern (see Fig. 2). Here, we describe the two aircraft and the data processing steps undertaken to prepare the dataset for publication.

### DataHawk2

**Fig. 3:**
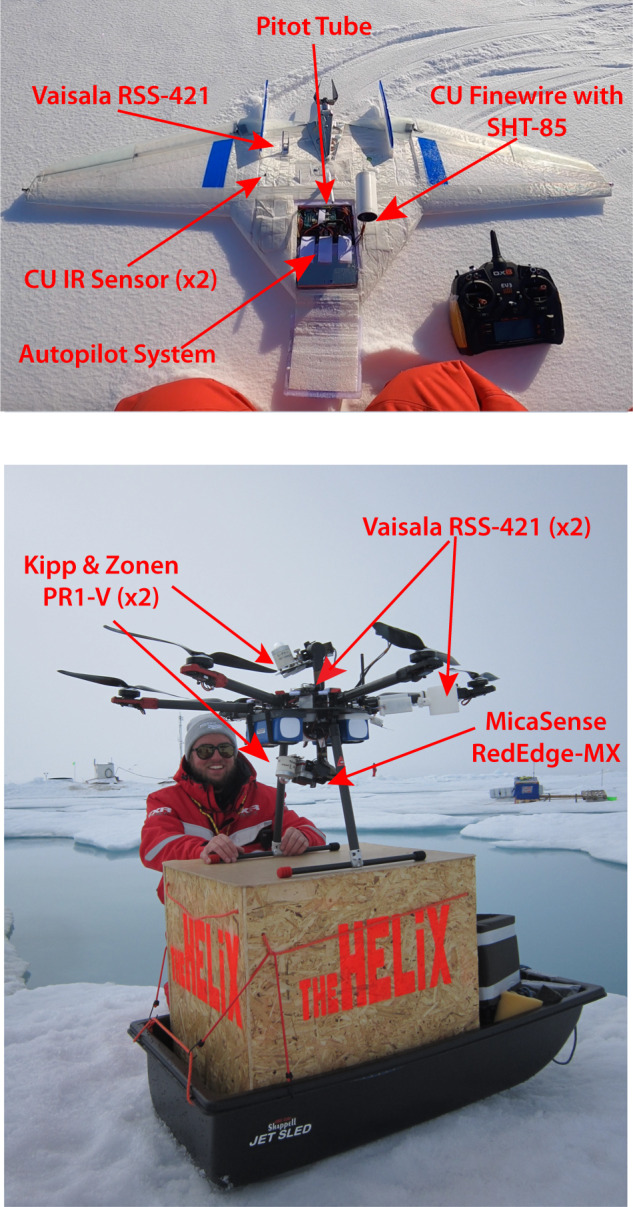
The DataHawk2 and HELiX UAS, as seen on the ice during MOSAiC. Primary measurement and aircraft systems are labeled in red text for both platforms. The HELiX photo (bottom) also shows the sled and box used to transport HELiX from *Polarstern* to the ice (photos: Gina Jozef and Radiance Calmer). Informed consent to publish individual likeness was obtained.

The DataHawk2 sUAS^20^ (Fig. 3 top) is a single-motor fixed wing aircraft with a wingspan of 1.3 m and total weight of approximately 1.8 kg. The system is powered by lithium polymer batteries, supporting a flight endurance of approximately 45 minutes (less at cold temperatures). The aircraft is controlled by a custom-designed autopilot system, or via a remote-control handset, and communicates with a ground station via a 2.4 GHz telemetry radio that relays some avionics and sensor data to the ground station for real-time display and enables supervisory control of flight parameters via operator commands.

For MOSAiC, the DataHawk2 was equipped with a variety of sensors to measure atmospheric state, aircraft attitude, and surface and sky infrared (IR) temperature. Measurements from these sensors were aligned in time to a common sampling clock. Raw measurements are saved to an on-board SD card at various sample rates (800 Hz, 100 Hz, 10 Hz, 5 Hz – sensor dependent), in blocks time-stamped by the processor clock. The 5 Hz blocks contain GPS time-of-week (TOW), which is subsequently used to time-synchronize all the data to GPS TOW in post-flight processing.

Aircraft position is measured by a single-frequency GPS receiver as well as a pressure-altimeter. The altimeter is more accurate on short timescales than GPS in estimating vertical position but suffers from long-term drift due to temperature and background pressure variation. The pressure and GPS signals are combined in post flight calibration, using a first order least-squares fitting procedure, to remove this drift for more accurate altitude measurements. The aircraft also carries a pitot static probe to measure airspeed as required for aircraft control and wind estimation. To correct the airspeed measured by the onboard pitot, a series of steps are undertaken. First, the launch time is determined, and the pitot pressure values just prior to launch are averaged to find a zero-airspeed value. Once this is completed a pitot calibration gain factor is applied, as determined from previous wind tunnel testing, and a density factor is calculated to allow for calibration of the pitot airspeed to the median GPS velocity to derive the final aircraft airspeed.

Aircraft attitude is estimated by direct integration of a high-rate 3-axis Micro-Electromechanical Systems (MEMS) rate gyro at 100 Hz, corrected for drift using high-rate 3-axis accelerometer and magnetometer sensors together with lower-rate GPS velocity and “moving baseline” differential GPS measurements of the wing tip-to-tip vector. Since the magnetic field vector is nearly vertical in high-latitude operations, use of the differential GPS wing tip vector rather than the gravity vector provides a more robust drift correction. Aircraft attitude is used in flight control for the elevation and bank inner loops, and for the compass heading to track a wind-aware guidance algorithm that improves GPS course heading tracking in high winds. Attitude is also used in estimation of horizontal wind, discussed below.

For measuring the thermodynamic state of the atmosphere, this includes a Vaisala RSS-421 pressure, temperature, and humidity (PTH) sensor suite. This sensor was situated on top of the airframe, on the starboard side of the airframe body in front of the tail fin. This positioning extends the sensor into the streamflow passing over the aircraft, which is white to reduce the potential for heating of the airframe and its influence on RSS-421 measurements. The RSS-421 is like Vaisala radiosonde (RS-41) sensors and identical to the Vaisala dropsonde system (RD-41). The RSS-421’s platinum resistive temperature sensor provides 0.01 °C resolution and a measurement repeatability of 0.1 °C, with a 0.5 s response time. The capacitive silicon pressure sensor features a 0.01 hPa resolution and repeatability of 0.4 hPa. The system’s thin-film capacitive relative humidity sensor actively monitors and corrects for sensor temperature, and offers a resolution of 0.1% RH, repeatability of 2% RH and a temperature-dependent response time ranging from 0.3 s (at 20 °C) to 10 s (at −40 °C). The RSS-421 sensors are not necessarily designed as multi-use sensors. However, through collaboration with colleagues at the National Center for Atmospheric Research, these sensors were equipped with a regeneration process to keep the relative humidity measurements functioning optimally. RSS-421 sensors on the DataHawk2 were replaced somewhat regularly during leg three, with an average time of use of 1.2 hours per sensor. Sensors were replaced less frequently on leg four, with only one exchange after 7.6 hours of use.

In addition to the RSS-421, the DataHawk2 also carried a finewire array, designed and developed by the University of Colorado. This sensor system consists of a shaded sensor array that is situated within a 2.54 cm diameter tube, which is mounted on top of the aircraft, on the port side of the battery and electronics hatch in front of the tail fin. The sensor array includes a pair of 5 µm diameter platinum wires extending over a 2 mm length, suspended in the free stream by supporting prongs, and a Sensiron SHT-85 sensor. One of the finewires is operated as a coldwire thermometer, with a regulated 1 °C overheating relative to the surrounding environment. The second wire is set up as a hotwire anemometer, with 100 °C overheating to the ambient temperature. These two wires have a thermal time constant of 0.5 ms under 15 m s^−1^ airflow. A custom-developed electronics module converts the change in wire resistance due to velocity or temperature variability to amplified voltages. Resolution of the raw time series are 8.3 × 10^−5 ^m s^−1^ for the hotwire and 1.3 × 10^−4 ^K for the coldwire. The sensor system supports sampling up to 800 Hz, enabling measurement of turbulent fluctuations in velocity and temperature at rates up to 400 Hz. For MOSAiC, these outputs were logged at 800 Hz on the DataHawk2’s SD card, which is equivalent to a 1.75 cm minimum sampling length scale at the DataHawk2 typical cruise airspeed of 14 m s^−1^. Finally, the SHT-85 sensor integrated into the finewire sensor is mounted inside the tube behind the finewires and has a stated temperature accuracy of 0.1 °C (from 20–50 °C) and a repeatability of 0.08 C, while the humidity sensor has a stated accuracy of 1.5% RH and a repeatability of 0.15% RH.

**Fig. 4:**
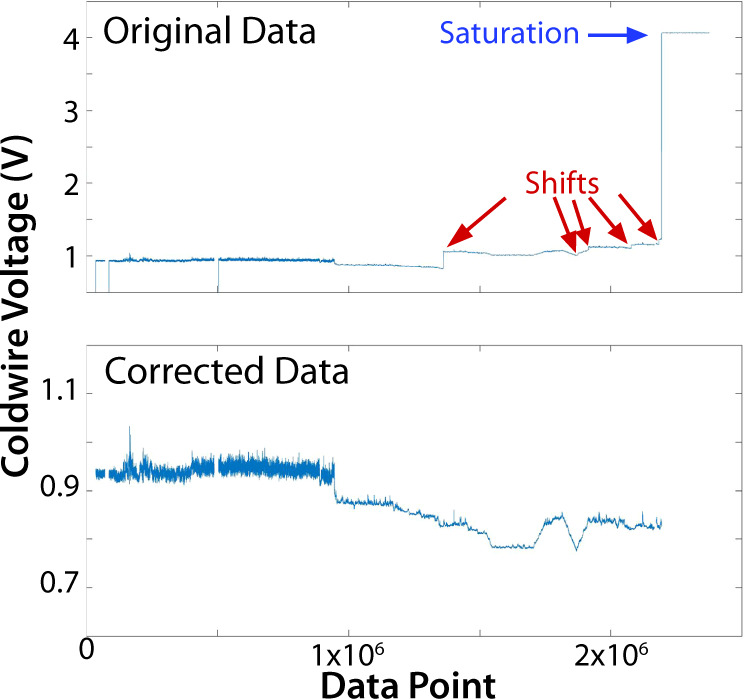
An illustration of the DataHawk2 coldwire data corrections applied. The top panel shows raw coldwire voltage data from a MOSAiC DataHawk2 flight. Notably, there are several voltage increases (red arrows) and toward the end of the flight the wire is broken and the signal saturates (blue arrow). The bottom panel shows the corrected coldwire voltage, after processing.

To convert coldwire voltage to air temperature, a calibration is applied using temperatures measured by the SHT-85 sensor. The SHT-85 sensor is positioned approximately 2 cm behind the two finewires, with an angular offset from the hotwire to ensure that the heating associated with that wire is not detected by the SHT-85. In the calibration process, a best fit linear regression is calculated between the SHT-reported temperature and coldwire voltage over the full temperature range of a given flight. The slope of this line is typically around 16 °C/V, but the offset can vary flight by flight. Applying this linear regression to the coldwire voltage time series produces a time series of associated temperature values in °C. In addition to fluctuations in the coldwire voltage resulting from turbulent fluctuations in atmospheric temperature (~1e-3 V), some flights also saw abrupt increases in coldwire voltage resulting from fretting of the sensor prongs in their sockets during flight (Fig. 4). These increases are not the result of atmospheric temperature changes and were therefore removed in post-processing to allow for accurate calibration of the coldwire sensor. To do this, coldwire increases were manually identified through analysis of the rate of change of the coldwire voltage. For each identified increase, the magnitude of the voltage change is subtracted from all subsequent coldwire voltages prior to calibration against the SHT-85 sensor. Increases in the coldwire voltage were observed in 26 flights, and in each of these flights, there were at most 9 increases throughout the flight. In addition to these increases, the fragile wires can be broken by airborne ice crystals, causing the coldwire signal to become saturated (> = 4 V) for the remainder of the flight (Fig. 4 near the end of the flight). Once the coldwire is broken, it no longer provides meaningful measurements, so saturated coldwire values are not included in the data points used for coldwire calibration. Breakage of the coldwire at some point throughout the flight was observed in 18 cases. Additionally, saturated coldwire voltages and the corresponding coldwire temperatures were replaced with “Not A Number (NaN)” values in the time series after calibration. The bottom panel of Fig. 4 shows the coldwire voltage time series after the jump and saturation removal processes have been implemented.

**Fig. 5:**
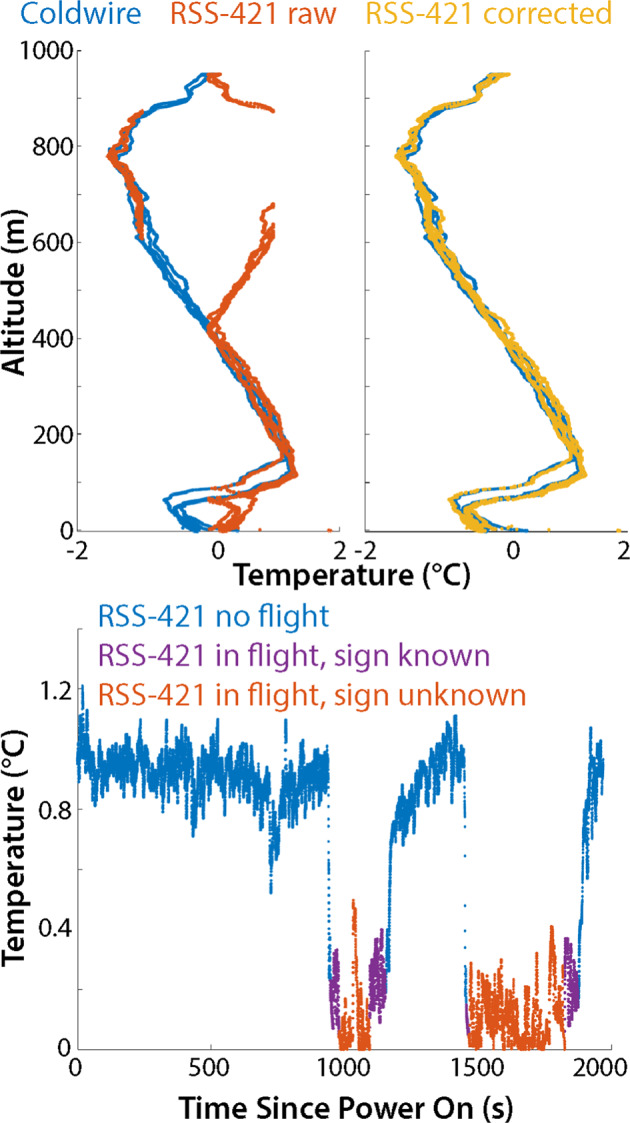
Examples of the RSS-421 sensor corrections applied to the DataHawk2 and HELiX datasets. In the DataHawk2 figure (top), the left side shows the coldwire (blue) and the uncorrected RSS-421 (red) temperature profiles, clearly illustrating the logging problem experienced between −1 and 0 °C. On the right is the same coldwire profile (blue), along with the corrected RSS-421 values (yellow). The HELiX figure (bottom) shows the temperature time series, including data points collected while the HELiX was not in flight or during take-off and touch down (blue points), data points known to be positive (purple) and data points of uncertain sign (red) due to their proximity to 0 °C.

The software used to interface the RSS-421 sensors with the DataHawk2’s onboard data logger included a bug that caused inversion of temperatures between 0 and −1 °C, resulting in the recording of positive temperature values in this temperature range. During leg 4, observations often covered the temperature range around freezing (−1 to 1 °C), which means that this software issue introduced uncertainty on the true temperature values observed. To correct for this issue, post-processing has been applied that leverages the multiple temperature measurements on board of the sUAS. This correction starts with a comparison between RSS-421 temperatures and those from the coldwire (which is calibrated using the SHT-85), or with those from the SHT-85 in cases where the coldwire was found to be inoperable. Because of its fast response time, the coldwire-derived temperature was the primary point of comparison used to assess whether RSS-421 temperatures between 0 and 1 °C were the correct sign, except for flights where the coldwire was broken, as described in the previous paragraph. For the 14 flights that saw a broken coldwire, temperatures from the SHT-85 sensor were used for this post processing evaluation. To conduct this correction, first data from all three temperature sensors are linearly interpolated to a common 5 Hz time clock. Then times are identified where the RSS-421 temperature was between 0 and 1 °C and the coldwire or SHT temperature is negative. As a first step, the sign of those data points is converted to make the RSS-421 temperatures negative. Because the primary measurements used to derive temperature from each of the sensors are independent (note that the SHT-85 is used to calibrate the coldwire temperature values, introducing some level of co-dependence), differences in temperature measurements on the order of 0.1 °C can readily occur. Therefore, additional evaluation is conducted to ensure that the RSS-421 data points closest to 0 °C were correctly modified. This includes a manual evaluation of measurement continuity around 0 °C. An example of this correction is shown in Fig. 5 (top), where the RSS-421 temperatures are initially of opposite sign in comparison to coldwire temperatures for some part of the DataHawk2 profile. The correction reconstructs the continuity of temperature measurements.

Wind sensing from fixed-wing UAS is a complex problem, particularly in gusty environments. For example, when the wind speed approaches the airspeed of the aircraft, tracking a GPS-referenced flight path becomes difficult due to the great disparity in ground speeds necessary on the up- and downwind legs. This requires the aircraft to turn rapidly on the downwind leg, requiring a large bank angle and loss of lift. This produces rapid changes in aircraft attitude that are difficult to estimate accurately with sensors that have different time constants such as the differential GPS and MEMS magnetometer deployed on the DataHawk2. Also, the angle of airflow over the airframe can change rapidly in these periods, and the DataHawk’s weathervaning is less accurate during these extreme maneuvers. This problem adversely affects wind estimation techniques as the DataHawk2 is not equipped with sensors to measure angle of attack and sideslip.

Nonetheless, some information on wind state can be derived from the sensor system on board the DataHawk2, and for the MOSAiC dataset two different techniques are applied to estimate horizontal winds. The first technique follows methods laid out in the literature^21^, with true airspeed calculated using information from the pitot probe and onboard thermodynamic sensors, and aircraft attitude information coming from the GPS system and inertial measurement unit (IMU). As angle of attack and sideslip are not measured by the DataHawk2, these are assumed to always be zero which translates into an expectation that the aircraft always points into the relative wind direction at the aircraft. This assumption results in unquantified errors in derivation of the turbulence characteristics of the wind. With this information, three additional corrections are applied to the dataset, including a time-lag correction that aims to account for differences in the time constants of the GPS system and the onboard IMU, a TAS correction that eliminates errors in the measurement of TAS, and a correction for potential angular offsets between the aircraft, IMU, and pitot probe. All these corrections are applied as minimizations that look to reduce the overall variance in the horizontal wind components, as TAS and misalignment problems will increase variability in these quantities over the scale of orbital flight. This minimization can only support corrections to the level where system-induced errors are larger than the natural variability found in the atmosphere at any given point in time over the length of an orbit or racetrack.

The second wind estimation technique attempts to remove one source of error that is caused by differing sensor time constants. Here, a horizontal wind vector is found that satisfies the wind triangle relating measured GPS velocity and relative wind, where only the magnitude of relative wind is used. This requires the horizontal wind vector at each instant of time to lie on a circle of known center and radius^22^. Previous wind estimates are updated by projecting onto this circle, in a direction given by the aircraft compass heading. The geometry of this projection makes the resulting wind estimate relatively insensitive to errors in compass heading attitude.

In addition to the sensors deployed to measure atmospheric thermodynamic state, the DataHawk also carried customized, fast-response (15 ms time constant) IR sensors based upon the Semitec 10TP581T thermopile. These sensors include a silicon window that limits incoming radiation to >5 um wavelength and has a half-power field of view of 90 degrees. One IR sensor is installed on the dorsal surface of the airframe (sky-facing in level flight), and one is installed on the ventral surface (surface-facing in level flight). The optical thermopile signal is amplified and combined with the integral thermistor signal to reduce the influence of sensor case temperature fluctuations, and the resulting signal is further calibrated to produce an approximate brightness temperature of the area in view of the sensor. Because these sensors are not thoroughly calibrated, they are primarily used to identify variations in surface temperature (e.g., resulting from leads or melt ponds) and sky temperatures (resulting from cloud cover) to allow data users to correlate these features with other measurements.

### HELiX

The HELiX sUAS (Fig. 3, bottom) is a hexacopter aircraft with a diameter of approximately 1 m (1.4 m including propellers), 61 cm height, and total weight of approximately 12 kg. The system is powered by lithium polymer batteries, supporting a flight endurance of up to 25 minutes (less at cold temperatures). The aircraft is controlled by the ArduCopter flight controller running on a PixHawk-based CubeBlack autopilot system, with manual inputs coming from a remote-control handset, and communication with a ground station via a 2.4 GHz telemetry radio. MOSAiC represented the first field deployment for the HELiX system.

As with the DataHawk2, the HELiX was outfitted with a variety of sensors to measure atmospheric and surface conditions, as well as the aircraft and gimbal orientation and attitude. All data were logged using a custom data logging system developed by the University of Colorado called FlexLogger. The FlexLogger combines multiple high-rate data streams into a common log format with synchronized clock. The hardware is based on the PRJC Teensy 3.5 with an ARM Cortex-M4 running at 120 MHz, and custom firmware. The design enables rapid driver development for a variety of sensors with output bandwidth up to 1 kHz. Data is time stamped using a common clock to 1 ms, which is synchronized to a GPS PPS signal. Binary data is stored to an onboard SD card and post-processed into NetCDF and Matlab formats. The synchronized clock time is logged independently with each data packet to allow for accurate synchronization of variables in post processing to a common 10 Hz clock.

Aircraft attitude and position was measured using the CubeBlack autopilot system. At the completion of the MOSAiC sampling period, it was noted that there were often differences in the altitudes recorded at take-off and landing positions, and that the take-off and landing altitudes were often significantly different than zero. Because the aircraft was, with the exception of three flights, operated from the sea ice surface, the altitudes at take-off and landing should always have been within approximately 1 m of mean sea level, and we attribute these offsets to challenges faced by the autopilot’s GPS receiver at high latitudes, where the elevation angles of GPS satellites are often small, and to drift in atmospheric pressure over the time of the flight as both pressure and GPS altitude are used in calculating aircraft altitude. To correct for these errors, a linear trend calculated between the take-off and landing time points was calculated and subtracted from the timeseries to account for the difference in altitude at those two points and make them consistent. Then, all altitudes are corrected so that the altitude values at take-off and landing are set to 0 m. For HELiX files that included two separate flights with a landing in between (FL02, FL04, FL05), a linear trend is calculated and removed for each independent flight segment. Finally, for the three flights that were carried out from the helideck of the Polarstern (FL32, FL33, FL34), take-off and landing altitudes were found to differ between flights, varying from 11.5 m (FL33) to 20.5 m (FL34). To be consistent between the three flights, the intermediate value of 13.8 m (FL32) has been selected to represent the take-off and landing altitude from the helideck. The attitude data and information from the autopilot are used together to produce a “flight flag” for each flight. This flag was designed to provide information on whether the aircraft is in flight, with the flag set to 0 when the system is on but still on the ground, set to 1 when the copter is armed and, in the take-off or landing phase, and set to 2 when the relative altitude is higher than 1 m above the take-off surface.

To observe atmospheric thermodynamic conditions, the HELiX carried a pair of Vaisala RSS-421 sensors (same as the DataHawk2). As with the DataHawk2, the RSS-421 sensors were replaced occasionally during leg four. In total, nine sensor replacement were conducted, with replaced sensors generally supporting less than one hour of flight time. These sensors were mounted in custom 3D-printed plastic housings on arms 1 and 3 of the HELiX and were logged by the FlexLogger at 5 Hz. The housings are designed in such a way that the temperature and humidity sensors are shielded from direct sunlight while allowing for significant ventilation from the downwash of the HELiX propellers. The RSS-421 pressure sensor is not exposed to this downwash regime, limiting the impact of the propeller-driven airflow on sensor pressure measurements. The software problem impacting RSS-421 temperature measurements between 0 and 1 °C discussed in the DataHawk2 section above was also a problem for the HELiX. Correction of this issue was more challenging for HELiX because the temperature close to the sea ice surface was very often around 0 °C, and the aircraft was often hovering or flying between the surface and 20 m altitude. Also, because the aircraft was generally not profiling, it was more difficult to understand whether the sensor was correct or incorrect when providing temperature estimates between 0 and 1 °C. To correct for this issue, additional steps are taken to identify points within this range. Points that cannot be erroneous due to the progression of temperature in the timeseries are left as is. For example, points that connect in a time series between 0.05 °C and greater than 1 °C are known to be positive because they continuously increase to greater than 1 °C. Similarly, the sign is flipped to be negative for points that connect 0.05 °C and less than −1 °C. Given this logic, there are very few points for which the sign of the temperature reading can’t be accurately determined. To ensure transparency to the data user, quality control flag value was added to indicate when reported temperatures could be either positive or negative. An example of the implementation of this flagging procedure is shown in Fig. 5 (bottom).

In addition to the RSS-421 temperature correction, a variety of processing steps were undertaken to derive a quality-controlled data product from that sensor. First, to provide data at a unified 10 Hz sample rate, a linear interpolation is used to map the temperature, pressure, and relative humidity from the RSS-421 to a 10 Hz clock uniformly used across all the sensors. For temperature and humidity, physical limits (<−30 °C or >30 °C for temperature, <0% or >150% for RH) were used to search for outlying (erroneous) data points. Additionally, temperature, pressure and relative humidity values falling outside of five standard deviations for the entire flight’s values are also identified as outliers. Because these outlying values generally represent time segments of <1 s, they are replaced with linearly interpolated data.

For each variable from the RSS-421 sensors, a quality control (QC) flag was created. This flag is set to be either 0 or 1 for pressure and relative humidity and 0, 1 or 2 for temperature. For all sensors, the flags are 0 when measurements are deemed to be reliable and 1 when the platform is on the ground and during take-off and landing (altitude <1 m AGL) or when values are reported as “Not a Number (NaN)”. The relative humidity flags are set to 1 when values are reported to be less than 0% or more than 102%, and pressure flags are set to 1 when pressures are reported to be outside of the range between 890 and 1020 hPa. Additionally, temperature flags are set to 2 when there is uncertainty about the sign of the measurement, as described above. Table 1 summarizes the flag values with the associated criteria.

**Table 1:** A summary of the quality control flag values for the RSS-421 sensors on the HELiX.

	In-flight, and reliable data	On ground or take-off/landing phase (alt <1 m)	NaN values, regardless of stage of flight	Outside of physical boundaries	Uncertain temperature sign between −1 and 1 °C
Pressure	0	1	1	1	N/A
RH	0	1	1	1	N/A
Temperature	0	1	1	N/A	2

Physical boundaries set for the pressure measurement are 980–1020 hPa, and physical boundaries set for the RH measurement are 0–102%.

To provide a common UTC time stamp for HELiX data GPS Time of Week (TOW) from the CubeBlack autopilot was used. Because the GPS TOW was not logged on the FlexLogger, pressure values from the RSS-421 and CubeBlack autopilot (both logged on the FlexLogger) were used to synchronize the timestamp to the rest of the FlexLogger data. To be consistent with other archived datasets, time was calculated as two independent quantities. These include the base time since Epoch (seconds since 1970-01-01T00:00:00.00) and the time offset from base time (in seconds).

Additionally, the HELiX was equipped with a pair of customized Kipp and Zonen PR1 pyranometers to measure hemispheric irradiance. These sensors have a spectral range between 310–2700 nm, and a fast response time (<0.2 s at 95%). They feature a temperature response of less than 1% for temperatures between −20 and 50 °C, and a non-linearity of <0.3% between 100–1000 W m^−2^. These sensors were mounted on active 2-axis gimbals mounted to the top and bottom of the HELiX to always keep the sensors perpendicular to the horizon.

**Fig. 6:**
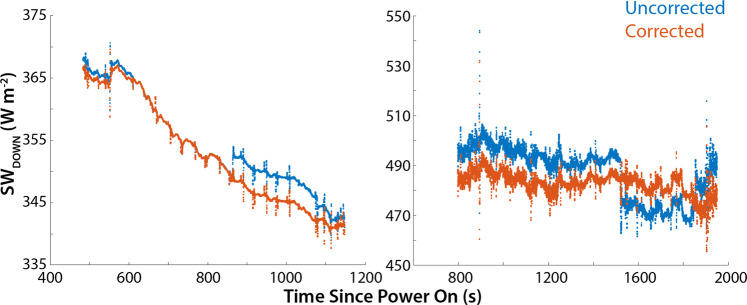
Examples of the correction applied to the Kipp & Zonen PR1-V downwelling shortwave irradiance data, collected on two days with limited cloud cover. The step change seen in the uncorrected data at approximately 875 s (left) and 1520 s (right) are the combined result of a change in the HELiX yaw angle and slight calibration offset in the gimbal supporting the upward-looking sensor.

Pyranometer data are quality controlled as well, with data points outside of the physical measurement range removed. Irradiance values lower than 0 W m^−2^ and higher than 800 W m^−2^ are replaced with NaN values. Furthermore, a QC flag is created, which is set to 1 when the system is on the ground and during the take-off/landing phase and 0 during the flight. The quality of the downwelling data is sensitive to sensor tilt angle in conditions with significant amounts of direct irradiance, and even a small tilt of the platform of less than 5° can cause error of tens of W m^−2^ in SWD measurements^23^. To evaluate the gimbal calibration, we leveraged data from two flights with limited cloud cover. In these data, downwelling shortwave radiation (SWD) showed a step in the measurement time series associated with a change of UAS yaw angle, indicating a slight misalignment of the gimbal system. To correct for this offset, methods previously applied to radiation data^24^ are implemented. Specifically, Equation (4) from the cited literature is used to calculate the corrected SWD for these two flights (see Fig. 6). As HELiX does not provide measurement of the direct and diffuse downwelling shortwave radiation components, the ratio K = Diffuse/Direct SWD is approximated to 0.1 for the clear sky flight (FL19) and is approximated to be 1 for cloudy conditions encountered during FL14^25^.

The HELiX sUAS was also equipped with a gimbal-mounted multispectral camera, the five-band RedEdge-MX, that has the following bands: blue (475 nm center, 32 nm bandwidth), green (560 nm center, 27 nm bandwidth), red (668 nm center, 14 nm bandwidth), red-edge (717 nm center, 12 nm bandwidth), and near-infrared (842 nm center, 57 nm bandwidth). This camera was used to take images of the surface overflown by the copter during all flight types: traditional single altitude grid patterns, low level hovers over surface features, and vertical profiles. During grid flights the camera was configured to use GPS position to trigger in a manner to collect images with 75% along-track overlap. For the hover and profiling flights, the camera was set up to trigger at 1 Hz. The first two flight patterns are well suited to producing orthomosaics, albeit with additional processing steps for the low-level hover flight type. The profile is not well suited to orthomosaic production, but the images can still be used to create a video of the flight that can be used to see what the copter is over and/or has in its field of view at any given moment.

Due to the significant drift of the ice flow over which the copter was flown, images taken with the RedEdge-MX needed to be transformed from the earth-relative GPS coordinate system to an ice-floe relative coordinate system to produce high quality orthomosaics. This was done by subtracting the change in GPS-derived position of an 11 m micrometeorological tower also positioned near *Polarstern* at a location referred to as Met City Tower (fixed relative to the ice floe) over the course of a flight from the GPS location tagged with the images. After this transformation was complete, testing was done with the Pix4D software package (https://www.pix4d.com) that determined that adding in the HELiX’s autopilot heading improved orthomosaic quality. Pix4D was then run using a modified version of the AG Multispectral template, along with the transformed image positions and orientations.

**Fig. 7:**
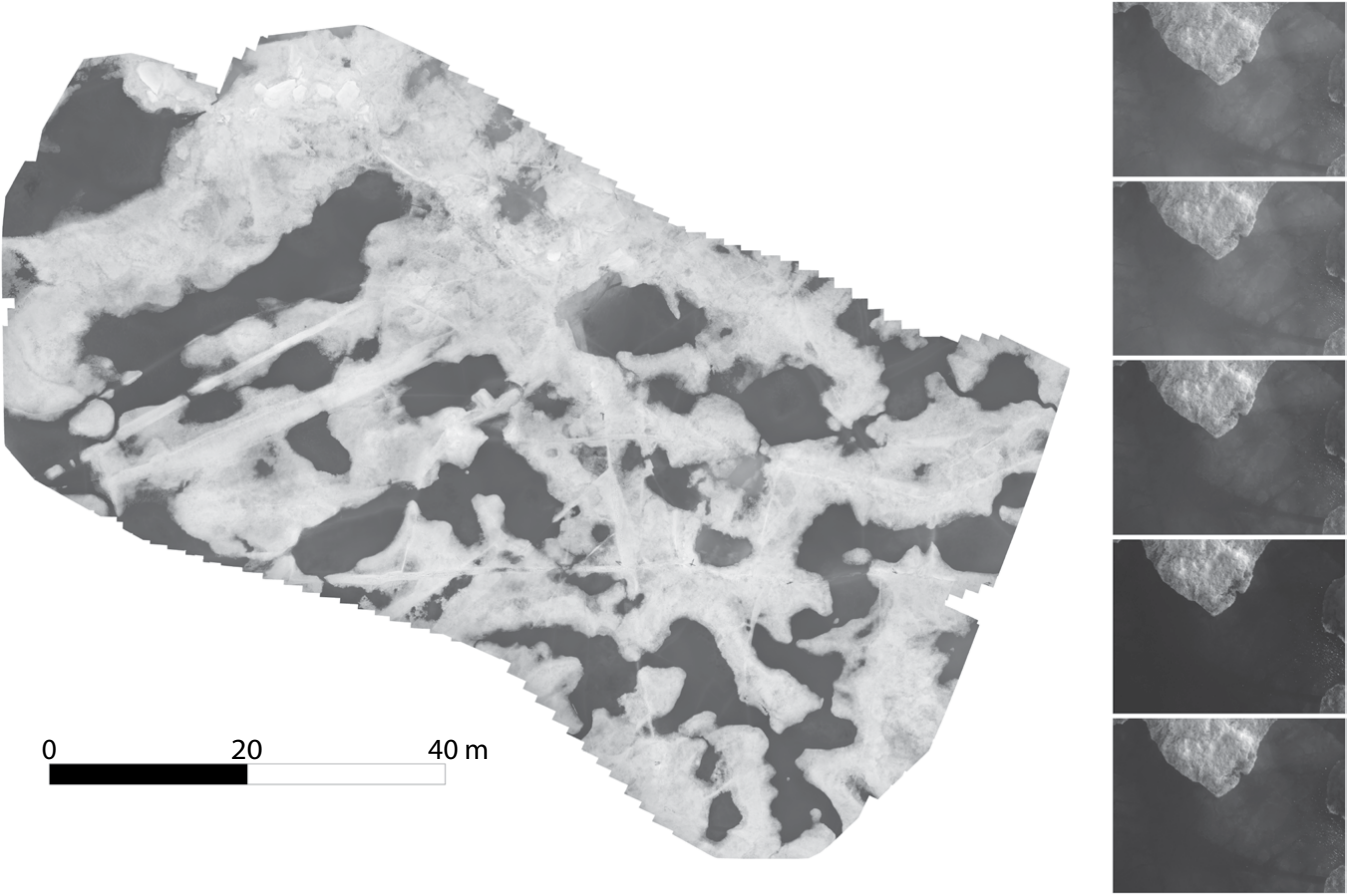
Example imagery from the HELiX multispectral camera. At left, an orthomosaic produced using blue band multispectral images from the HELiX RedEdge-MX camera system, joined together using Pix4D software. At right, a set of images showing each band of the RedEdge-MX, from top to bottom: blue, green, red, red edge, near-infrared.

Every flight was processed using Pix4D, which produced three data products for each of the five RedEdge-MX channels. The different channels help highlight different features in the ice (see Fig. 7). First, an orthomosaic of the images taken during a flight was produced for each band, resulting in five composite images of the area overflown. Second, a reflectance map for each band was produced, with the pixel values corrected by Pix4D using images of the RedEdge-MX’s calibration reflectance panel taken before and after each flight. Third, a colorized index map was produced as an example of what can be done with further analysis; in this case, five colors were assigned to equal surface area bins to colorize each band’s index map. With further analysis, the number of bins and ranges for each can be changed in Pix4D to highlight specific features. These products were produced for the whole area overflown on grid flights, for a sample area of interest on low-level hover flights, and for images no less than 50% the maximum altitude (a recommendation for successful processing by Pix4D) on profile flights. It should be noted that Pix4D struggled to produce data products over areas consisting primarily of water; the lack of discernible features makes it difficult to match features between images and stitch them into an orthomosaic. This leads to some missing images in grid flights, or a lack of ability to create orthomosaics in areas of interest that were predominately melt ponds or open ocean. Lastly, videos for the profile flights have been produced with an altitude and flight time overlay, so one can, for example, see what the copter was overflying for a given albedo value.

## Data Records

### DataHawk2 data

There are three levels of DataHawk2 data archived at the NSF Arctic Data Center^26–28^. Raw data files are produced and archived in Matlab format, while level A1 and B1 files are available in NetCDF format. Level A1 data are time-aligned but provided at the native resolution logged on board the aircraft’s SD card. Level B1 data have been time-synchronized, quality controlled, and corrected as described in the methods section above, and are all reported on a common 10 Hz clock. Additionally, the B1 data include a ‘Flight_Flag’ variable to report when the aircraft is in flight, and data have been trimmed to only include the portion of the file between take-off and landing. File name conventions for the two datasets are similar, taking the form CU-DH2_YYYYMMDD_hhmmss_DL.nc, where YYYYMMDD_hhmmss is the year, month, day and hour, minute and second of the flight (UTC), while DL is the data level (either A1 or B1).

**Fig. 8:**
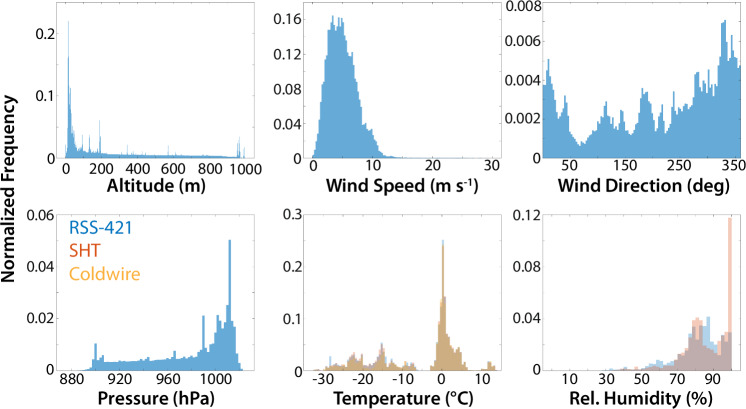
Histograms summarizing the range of values measured by the DataHawk2 during Legs 3 and 4 of MOSAiC. Included are (clockwise from top left) panels showing aircraft altitude, wind speed, wind direction, relative humidity, temperature, and pressure. Values observed by the RSS-421, SHT-85 and coldwire sensors are included in the bottom row.

The DataHawk2 was operated in two separate flight patterns during the MOSAiC project. The first, and most common pattern was an orbital profile extending from the ice surface to 1000 m or cloud base, whichever was lower. During a profiling flight, the center of the circle was often shifted to keep the aircraft overhead of the operators sitting on the drifting ice. The second flight pattern that was executed was a “racetrack” pattern where the aircraft was held at a constant altitude while sampling horizontally between two circles. This pattern was used to collect data on the spatial variability of thermodynamic properties over the ice surface, particularly over inhomogeneities in the surface such as leads. Together, these flight patterns captured a wide variety of different weather conditions, as shown in the histograms in Fig. 8. Temperatures sampled throughout the campaign ranged from approximately −35 to 15 °C, with much of the Leg 4 flight time conducted in conditions near 0 °C. Relative humidities were generally greater than 70% and wind speeds sampled by the DataHawk2 were generally less than 10 m s^−1^. The different sensors for temperature and relative humidity generally sampled consistent values, though some differences are evident between the Vaisala RSS-421 sampled RH values and those measured by the SHT-85. These differences could be due to a variety of factors, including challenges with the regeneration process of the RSS-421, as well as differences in the response times of the two sensors. Additional discussion on the measured humidity values is included in the Technical Validation section below.

Future work may extend the data products available from the DataHawk2 measurements. Specific products of interest include those targeting turbulent parameters, including the turbulence dissipation rate, temperature structure function, bulk Richardson number and Brunt-Vaisala frequency. These characteristics have been calculated previously using iterations of the DataHawk^29,30^. Additionally, there is hope that the wind estimation can be improved further, possibly requiring future data processing and development of a new wind product. Finally, the IR sensors on board the DataHawk2 are uncalibrated, and some limited efforts have been made to develop a calibration technique for these sensors that would allow the team to develop an IR brightness temperature product. However, these products would require a significant amount of additional effort and are not available at the current time.

### HELiX data

As with the DataHawk2, there are also three levels of HELiX data archived at the NSF Arctic Data Center^31–33^. As with the Datahawk2, the raw (A0) level data are recorded and provided in Matlab format, while the A1 and B1 data levels are available in NetCDF format. Level A1 data include raw data at the native resolutions logged by the FlexLogger. Level B1 data have been time-synchronized, quality controlled, and corrected as described in the methods section. Quality control flags have been added to the B1 data files to help users identify potentially erroneous data, again as described in the previous section. File name conventions for the two datasets are similar, taking the form MOSAiC_CU-HELIX_YYYYMMDD_hhmmss_DL.nc, where YYYYMMDD_hhmmss is the year, month, day and hour, minute and second of the flight (UTC), while DL is the data level (either A1 or B1). In addition to the data files, a separate dataset^34^ contains the imagery products, with the flight folders following the same naming convention as above, with the exception that DL is replaced with IMG. Each flight folder contains five orthomosaics (one for each camera band), five reflectance maps, and five colorized index maps.

**Fig. 9:**
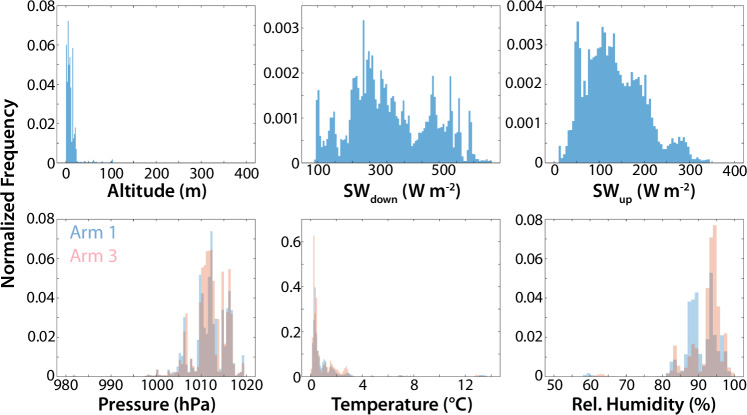
Histograms summarizing the range of values measured by the HELiX during Leg 4 of MOSAiC. Included are (clockwise from top left) panels showing aircraft altitude, downwelling and upwelling shortwave broadband irradiance, relative humidity, temperature, and pressure. Values observed by the RSS-421 sensors are included for both arms in the bottom row.

The HELiX was flown in three general flight patterns, including low-altitude (<20 m) surveys where the aircraft was operated in a grid pattern over the sea ice surface to map out surface properties, extended very low altitude (<5 m) loiter periods to measure albedo of a given surface type, and profile flights where the aircraft would start over a particular surface type and then climb to 100 m or more above the surface to evaluate the impact of scale aggregation on measured surface albedo. Histograms in Fig. 9 provide an overview of the HELiX measurements and flight altitudes for all flights conducted during leg 4 of the MOSAiC project. Most flight time was spent below **25 **m altitude, with the profiles discussed above extending up to altitudes between 100–400 m. This altitude coverage also stands out in the histogram of measured pressure values, with the variability in the pressure variable mainly resulting from changes to the local atmospheric pressure. Temperature and relative humidity were centered in the same range of values, between 0 and 4 °C and 80 and 100%, respectively for most of the flights conducted, which is not surprising for a campaign conducted close to the sea ice in the Arctic summer. Nevertheless, a few flights occurred during a warm air mass advection above the MOSAiC floe when the expedition reached the ice edge. High temperatures (~12 °C) and low relative humidity (60%) were measured during this period. The histograms reveal good agreement between the RSS-421 sensors on Arm 1 and Arm 3. However, some differences are present, particularly in the measured relative humidity, which could be due to the displacement direction of the HELiX or to challenges experienced with the conditioning of the onboard Vaisala RSS-421 sondes. Finally, the histograms of up- and downwelling irradiance provide perspective on the types of lighting conditions the HELiX was operated in, and the relative brightness of the surface during Leg 4.

## Technical Validation

To validate the quality of the data collected by the DataHawk2 and HELiX, several comparisons were completed with other sensor systems deployed during MOSAiC. These comparisons break down into evaluation of the thermodynamic sensors deployed on both platforms, and evaluation of the pyranometer/gimbal configuration in providing accurate measurements of down- and upwelling broadband shortwave irradiance. In this section we provide an overview of these comparisons, leveraging observations from three key sensor systems. This includes temperature, pressure, humidity, and wind observations from the radiosondes (Vaisala RS-41) launched from the *Polarstern* during MOSAiC at least 4x daily^35^. This also includes temperature and humidity measurements from Vaisala HMT 330 sensors on the meteorological tower at 2, 6 and 10 m and pressure observations from a Vaisala PTU 300 at 2 m, as well as temperature observations from a Vaisala HMT 330 and relative humidity and pressure observations from a Vaisala PTU 300, all at 2 m on the Atmospheric Surface Flux System^36^ (ASFS) sled deployed on the sea ice. Finally, it includes a comparison of HELiX-based measurements to up- and downwelling broadband irradiance from Hukseflux SR30 pyranometers positioned at 2 m on the ASFS and Eppley PSP pyranometers positioned at 1.5 m on the ARM radiation station at Met City^37–39^.

**Fig. 10:**
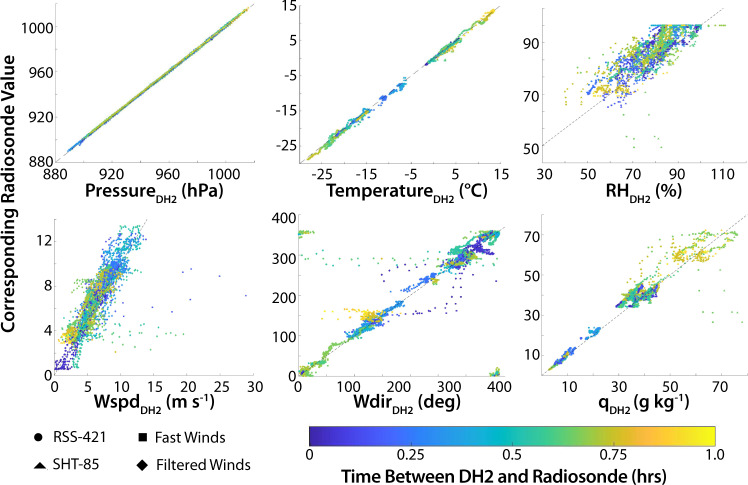
Scatter plots illustrate comparisons between the DataHawk2 observations and those from radiosondes deployed during MOSAiC. Included are comparisons of (clockwise from top left) pressure, temperature, relative humidity, specific humidity, wind direction, and wind speed. Color coding indicates the time difference between the DataHawk2 flight and the radiosonde at a given altitude. For temperature and humidity figures, symbols indicate comparisons for the RSS-421 (circles) and the SHT-85 (triangles).

Figure 10 illustrates comparisons between the DataHawk2 observations and those from radiosondes launched during MOSAiC. Included are comparisons of both the RSS-421 and SHT-85 sensors for thermodynamic quantities, and for the two wind estimates provided in the DataHawk2 dataset for wind speed and direction. As may be expected, pressure and temperature measured by the DataHawk2 compare very well with the radiosonde data. This illustrates that despite a consistent replacement schedule, there was little drift in the temperature and pressure measurements from the RSS-421s. The relative humidity values reported by the RSS-421 on the DataHawk2 are shown to be biased low. This is not a result of sensor performance, but rather the result of challenges that the team had with the regeneration process that should have been implemented daily. This regeneration process keeps the relative humidity sensor operating in an optimal fashion. However, after repeated sensor failures resulting from the regeneration process, and given an inability to re-supply the team with additional sensors, the decision was made to discontinue the process to preserve sensors. Users of the data are therefore advised to be cautious with the humidity data from the RSS-421 for this mission. The SHT-85 sensor shows closer agreement with the radiosonde, except for high (>85%) relative humidity values. However, these higher RH comparisons for the SHT-85 took place with a temporal difference of nearly one hour, allowing for the possibility of atmospheric changes being responsible for the observed differences. Similarly, the specific humidity comparison also shows a dry bias for the DataHawk2 sensors. Finally, a comparison of DataHawk2 and radiosonde winds generally shows agreement between the two platforms. Wind directions reported by the DataHawk2 generally fall in line with those from the radiosondes, though there are a couple of flight segments where the DataHawk2’s wind estimation algorithms seem to diverge, resulting in offsets in both wind speed and wind direction. Users are therefore advised to use the wind data cautiously, though we anticipate that qualitative evaluation of these data and quantitative averaged wind data should be valuable.

**Fig. 11:**
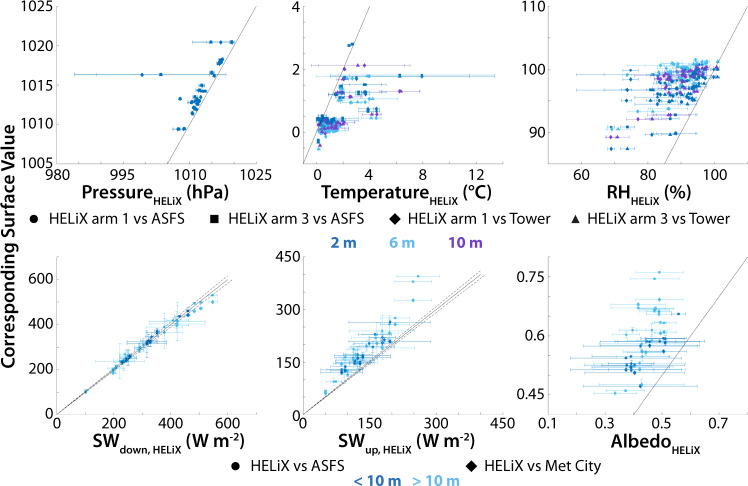
Scatter plots illustrate comparisons between the HELiX observations and those from other systems deployed during MOSAiC. Included are (clockwise from top left) panels showing air pressure, air temperature, air relative humidity, surface albedo, upwelling shortwave irradiance, and downwelling shortwave irradiance. Each panel has a variety of symbols to demonstrate comparisons to the ASFS or tower-based observations, as well as to demonstrate the comparisons as a function of altitude.

Figure 11 provides an overview of comparisons between HELiX-based observations and those from other systems deployed during the MOSAiC expedition. Each panel includes comparison to multiple surface-based systems and those comparisons are done across several altitudes, as indicated by the different symbols and colors. The variability across the sampling time window is shown by the bars extending out from the symbols, which represent mean values. For thermodynamic observations, the altitudes used for comparison are data points collected by the HELiX between 0 and 4 m (to 2 m surface observations), between 4 and 8 m (to 6 m surface observations) and between 8 and 12 m (to 10 m surface observations). For the shortwave irradiance observations, comparisons are done for times that the HELiX was below 10 m and above 10 m, because of the expected aggregation of surface features with altitude. Pressure measurements from the HELiX generally show a small low bias relative to those from the ASFS and met tower, on the order of approximately 1–3 hPa. This may be the result of having more data points between 2–4 m altitude than between 0–2 m altitude, or it could be connected to the use of rotor downwash for ventilation of the sensor system. Additional characterization of the observing system would be required to confirm this. Two flights show more significant low biases, resulting from a malfunctioning RSS-421 pressure sensor. These flights should be considered carefully by users (see quality control section). Temperature observations from the HELiX show a small but significant (1–2 °C) warm bias. This bias is difficult to explain but could be the result of heating of the RSS-421 from motor heat during flight. As with pressure, additional characterization of the system relative to a tower is required to say for sure whether these differences are the result of the system configuration, or different reasons. Finally, relative humidity data from HELiX are also shown to be biased low by 0–20%. These differences are the result of the RSS-421 sensors not being regularly re-conditioned during the flight campaign as required to keep the measurements within manufacturer specifications. Based on these comparisons, we note that thermodynamic observations from HELiX are given as indicative values, and users should carefully consider whether they are appropriate for scientific analysis.

In addition to evaluation of the thermodynamic variables, the bottom half of Fig. 11 provides a comparison of the shortwave broadband irradiance observations with those from the ASFS and surface-based observations in Met City. Of these, downwelling irradiance is expected to be the most directly comparable, though small changes in cloud properties or surrounding atmosphere can result in differences. The HELiX-observed downwelling shortwave irradiance is shown to agree very well with those observed by the ASFS and Met City instrumentation. Most data points fall within the range expected based on the manufacturer’s expected accuracy (dashed lines). The upwelling shortwave irradiance also agrees well in terms of the relative slope of the observed quantities but appears to show a low bias on the side of the HELiX. However, this difference is readily attributed to differences between the field of view of the sensors, with the surface-based sensors focused on a relatively small area from approximately 2 m above the ice surface, and the HELiX-based sensors generally observing a larger area at an altitude of 10 m or greater. Because the surface-based observations were likely positioned over solid ice with snow cover, they likely provide observations that are on the high side of the spatial distribution. However, the HELiX observations are likely to capture more of the variability, including influence of surface melt ponds or open leads, which would reduce the albedo observed by that platform. It is notable that the variability of most of the HELiX flights overlaps with the one-to-one line, and that observations collected by HELiX below 10 m tend to fall closer to that one-to-one line than those obtained at higher altitudes. In short, the observed upwelling irradiance is reduced relative to that observed over a snow-covered ice surface as the altitude of the sensors is increased due to the increased influence of water features on the ice surface. These differences in the upwelling irradiance also carry through to the albedo observed by the HELiX platform, which is generally lower than that observed by the surface observing systems.

## Usage Notes

The datasets described in the current document are available for public download and use. The authors would appreciate, but do not require, any potential data users to contact the lead author of this manuscript so that we are able to assist with any questions and are able to keep track of how the data collected are being used. Additionally, such outreach provides for an opportunity to share known issues with the dataset that may have come to light since the development of the current manuscript and allow for the sharing of software developed between the publication of this manuscript and the time of data usage. In this section we provide some perspective on the usage of the data and known considerations when using the DataHawk2 and HELiX datasets.

One primary consideration for using the DataHawk2 data is that care should be taken with any measurements obtained while the aircraft was not in flight. Due to internal heating of the aircraft systems and absorption of solar radiation by the fuselage, the sensors are likely measuring body effects when not ventilated by air moving over the platform. Additionally, the first 15 seconds of time after launch of the aircraft should also be treated with caution as sensors are adjusting to the increased airflow. A flight flag is provided to the user to help identify times when the data from a particular sensor may be impacted by a lack of airflow, and the user should make sure to leverage this flag. B1 files are interpolated to a common 10 Hz timestamp. Users should be aware that this interpolation did not include any anti-aliasing techniques, and aliasing is possible at higher frequencies. Therefore, these data should not be used to assess small scale turbulence in the atmosphere. Another consideration when using the DataHawk2 data is that wind estimation is a work in progress. As described above, two different estimates of the wind are provided, including one high-rate estimate that attempts to correct for airspeed and alignment offsets, and one filtered method that provides a smoothed version of the winds. Vertical winds are not provided due to the challenges associated with calculating these without an angle of attack sensor. Wind estimation becomes particularly challenging under dynamic flight conditions that may result in steep bank or elevation angles, or in very turbulent environments due to the likelihood of increased variability in aircraft angle of attack and sideslip (not measured on the DataHawk2). All wind data should be used with care, and any users of these data should recognize that they are likely not providing clean estimates of turbulence in the wind field.

Users of the HELiX data are strongly encouraged to pay attention to the quality control flags that are included with the dataset. The temperature and humidity measurements have been shown to have significant biases and are known to include some instances of particularly unreliable measurements. The causes for these sensor performance issues are not fully understood at the current time. Additionally, there are some flights where the aircraft was operated in such a way that heat from the platform or motors was detected by the RSS-421 sensors when flying in a specific direction. These instances are recognizable as step functions in the temperature and relative humidity measurement that are a function of the aircraft flight direction, and no quality control is applied to specifically identify these periods. It is therefore left to individual users to evaluate the possibility of contamination of the thermodynamic data because of this issue. As with the DataHawk2, users are advised to not use measurements collected while the aircraft is sitting on the ground because during those times the sensors are not ventilated. Finally, during some HELiX flights it was apparent that some condensation collected on the pyranometer sensor domes, though the comparison to the ASFS appears to indicate that this did not have significant effects on the measured irradiance.

For both platforms, it is important to recognize that the aircraft were operated in a highly dynamic environment. The ice pack was drifting underneath the aircraft, both of which operated using a GPS-supported navigation system. Therefore, it should be noted that the surface underneath the aircraft was constantly changing, and a given point on a flight pattern is not likely to be conducted over the same surface type as aircraft are orbiting or hovering. Additionally, this dynamic environment required the operators to constantly adjust the flight pattern to allow the aircraft to track their position, resulting in irregular flight patterns. Another part of the dynamic environment is the potential impacts of human activities in the flight area, and the potential impacts of the *Polarstern* and its cross-sectional area and thermal emissions on wind and temperature structure near the surface. The datasets described here were typically collected within 500 m of *Polarstern*, and therefore potentially subject to measuring the influence of the ship on the atmosphere. The dataset is currently not configured with any sort of indicator of the position of the ship during flight, and users are encouraged to download ship position information and use that along with wind information to evaluate whether measurements may be impacted by the presence of the *Polarstern*.

## Data Availability

The code used to produce and process these datasets was developed using Matlab. All of the routines used to develop the A1 and B1 data products are available in an open access Zenodo repository^40^.
