# Publisher Correction: Observing the Central Arctic Atmosphere and Surface with University of Colorado uncrewed aircraft systems

**DOI:** 10.1038/s41597-024-03954-1

**Published:** 2024-10-31

**Authors:** Gijs de Boer, Radiance Calmer, Gina Jozef, John J. Cassano, Jonathan Hamilton, Dale Lawrence, Steven Borenstein, Abhiram Doddi, Christopher Cox, Julia Schmale, Andreas Preußer, Brian Argrow

**Affiliations:** 1 Cooperative Institute for Research in Environmental Sciences, University of Colorado Boulder, Boulder, Colorado USA; 2 Physical Sciences Laboratory, National Oceanic and Atmospheric Administration, Boulder, Colorado USA; 3 Integrated Remote and In Situ Sensing, University of Colorado Boulder, Boulder, Colorado USA; 4 National Snow and Ice Data Center, University of Colorado Boulder, Boulder, Colorado USA; 5 Department of Atmospheric and Oceanic Sciences, University of Colorado Boulder, Boulder, Colorado USA; 6 Department of Aerospace Engineering Sciences, University of Colorado Boulder, Boulder, Colorado USA; 7 Extreme Environments Research Laboratory, École Polytechnique Fédérale de Lausanne, Sion, Switzerland; 8 Department of Environmental Meteorology, University of Trier, Trier, Germany; 9 Helmholtz Centre for Polar and Marine Research, Alfred-Wegener-Institute, Bremerhaven, Germany

**Keywords:** Atmospheric dynamics, Cryospheric science, Atmospheric dynamics, Cryospheric science

Correction to: *Scientific Data* 10.1038/s41597-022-01526-9, published online 23 July 2022

In the version of the article initially published, Figs. 8 and 9 were inadvertently swapped, and have now been amended in the HTML and PDF versions of the article.

